# Association Between Semaglutide or Tirzepatide Therapy and Residual Gastric Content: A Potential Danger During Upper Endoscopy

**DOI:** 10.7759/cureus.96771

**Published:** 2025-11-13

**Authors:** Jimmy Wen, Jose Puglisi, Eldo Frezza

**Affiliations:** 1 Physical Medicine and Rehabilitation, California Northstate University College of Medicine, Elk Grove, USA; 2 Biostatistics, California Northstate University College of Medicine, Elk Grove, USA; 3 Surgery, California Northstate University College of Medicine, Elk Grove, USA

**Keywords:** egd, esophagogastroduodenoscopy, residual gastric content, semaglutide, tirzepatide

## Abstract

Introduction

This study aims to evaluate the risk of residual gastric content (RGC) among patients taking semaglutide/tirzepatide undergoing an esophagogastroduodenoscopy (EGD).

Methods

A retrospective observational study was conducted from 2024 to 2025 for 144 patients who had been taking semaglutide/tirzepatide and were undergoing an EGD. The primary outcome was evaluating the presence of RGC, and the secondary outcomes were procedure-related complications. Statistical analysis included Fisher’s exact test, Spearman’s correlation, and Firth’s logistic regression.

Results

Among the 144 patients, 12 patients were taking semaglutide or tirzepatide for more than six months. Eleven of whom had RGC (seven semaglutide and four tirzepatide). No RGC was noted in the 132 patients who did not meet the inclusion criteria. Fischer’s exact test showed a significant association between GLP-1 usage for over six months and the presence of RGC (p<0.001). Spearman’s correlation found a moderate positive association between RGC and age, but a weak negative association with body mass index (BMI). Firth’s logistic regression further demonstrated a good model fit (pseudo-R^2^ of 0.943), but the individual predictors (glucagon-like peptide-1 (GLP-1) type, age, and BMI) were not statistically significant. There were no procedure-related complications, and the patients were discharged on the same day.

Conclusion

Semaglutide/tirzepatide for over six months resulted in RGC despite following the standard fasting protocol before EGD.

## Introduction

Semaglutide, a glucagon-like peptide receptor-1 receptor agonist (GLP-1 RA), is increasingly used to manage type 2 diabetes and obesity. Semaglutide mainly binds to plasma proteins with a half-life of one week and remains in circulation for five weeks [1]. While it has demonstrated efficacy in improving glycemic control and promoting weight loss, one of its known side effects is delayed gastric emptying, which can lead to gastroparesis [2]. Studies in humans have shown that GLP-1 infusion slows gastric emptying and increases both fasting and postprandial gastric volumes, reducing postprandial glycemia [3].

This delayed gastric emptying poses a risk during medical procedures such as esophagogastroduodenoscopy (EGD) and affects the absorption of concomitantly administered oral medications [2]. Thus, the American Society of Anesthesiologists (ASA) has established guidelines for patients taking GLP-1 RAs in which they recommend discontinuing the medication one day or one week prior, depending on whether the agent is a daily or weekly dosage [4]. Furthermore, if the patient has gastrointestinal (GI) adverse effects such as nausea, vomiting, or abdominal pain on the day of the procedure/surgery, canceling the case should be considered [4].

The literature indicates that diverse methods have been employed to measure the impact of GLP-1 drugs on gastric emptying, with most studies utilizing the acetaminophen absorption test [5]. Scintigraphy (for both solids and liquids) and acetaminophen absorption at 30 or 60 minutes have provided valid measures showing that GLP-1-related drugs delay gastric emptying [2]. This is associated with reduced glycemia and varying effects on food intake and appetite. GLP-1 agonists and analogs play a central role in managing type 2 diabetes mellitus (T2DM) and obesity, with long-acting formulations or prolonged use of short-acting ones potentially reducing gastric emptying due to tachyphylaxis [6]. Based on current reports, dual agonists targeting GLP-1 and another receptor (e.g., glucose-dependent insulinotropic polypeptide (GIP)) do not appear to affect gastric emptying [6].

In this study, we explore the potential dangers associated with performing an upper endoscopy in patients on semaglutide/tirzepatide therapy, emphasizing the risk of food residue in the stomach despite adherence to standard fasting protocols. Such residual gastric content (RGC) can increase the likelihood of complications such as pulmonary aspiration during the procedure, particularly under general anesthesia. The inspiration for this study came from observations in the senior author’s practice, where there was a noticeable increase in the number of patients with multiple food particles remaining in their stomachs compared to the previous year. To investigate this further, we decided to review the charts of patients who presented with food particles in their stomachs to identify any commonalities or potential contributing factors.

## Materials and methods

Study design

A retrospective observational study from 2024 to 2025 was conducted with patients taking semaglutide or tirzepatide who underwent an elective EGD for upper GI symptoms, mainly gastritis and reflux. Local approval for institutional review board (IRB) exemption was obtained at Colusa Medical Center from the Medical Executive Board. This study was conducted following the principles outlined in the Declaration of Helsinki. All data was de-identified to ensure patient confidentiality. Informed consent was obtained from the included participants.

Study population and procedure

Adult patients over 18 years who underwent EGD during the study period were identified using electronic medical records. The inclusion criteria were patients with type 2 diabetes and obesity, and the use of semaglutide (2.4 mg) or tirzepatide (2.5 mg) for greater than six months. Exclusion criteria included patients < 18 years old, history of gastroparesis or gastric obstruction, medications that delay gastric emptying, other GLP-1 agonists, incomplete data, and patients who did not undergo EGDs.

The institutional pre-procedural protocol prior to EGDs required patients to stop GLP-1 medications for three days before the procedure. Among these patients, we looked for signs of gastroparesis or any description of RGC in the stomach during the EGDs. RGC was defined as the presence of any documented food particles in the stomach. The patients were NPO for at least 12 hours and stopped taking semaglutide/tirzepatide 5-6 days before the procedure. The primary outcome was the detection of RGC, and the secondary outcome was procedure-related complications.

Statistical analysis

Fisher’s exact test was used to evaluate the association between GLP-1 RA usage duration (greater than versus less than six months) and the presence of residual gastric content (RGC). A two-sided p-value of <0.05 was considered statistically significant. Spearman’s rank correlation was employed to assess the relationship between RGC and continuous variables such as age and body mass index (BMI).

The sample size was determined from the number of eligible patients during this study period. Thus, no a priori sample size calculation was performed due to the retrospective observational nature of the study. Given the small sample size and potential for separation bias, Firth’s penalized logistic regression was utilized to model the likelihood of RGC based on GLP-1 RA type (semaglutide versus tirzepatide), age, and BMI. Model fit was evaluated using McFadden’s pseudo-R², a commonly used goodness-of-fit measure in logistic regression. All statistical analysis was calculated using the R software logistf package version 4.5 (R Foundation for Statistical Computing, Vienna, Austria).

## Results

**Figure 1:**
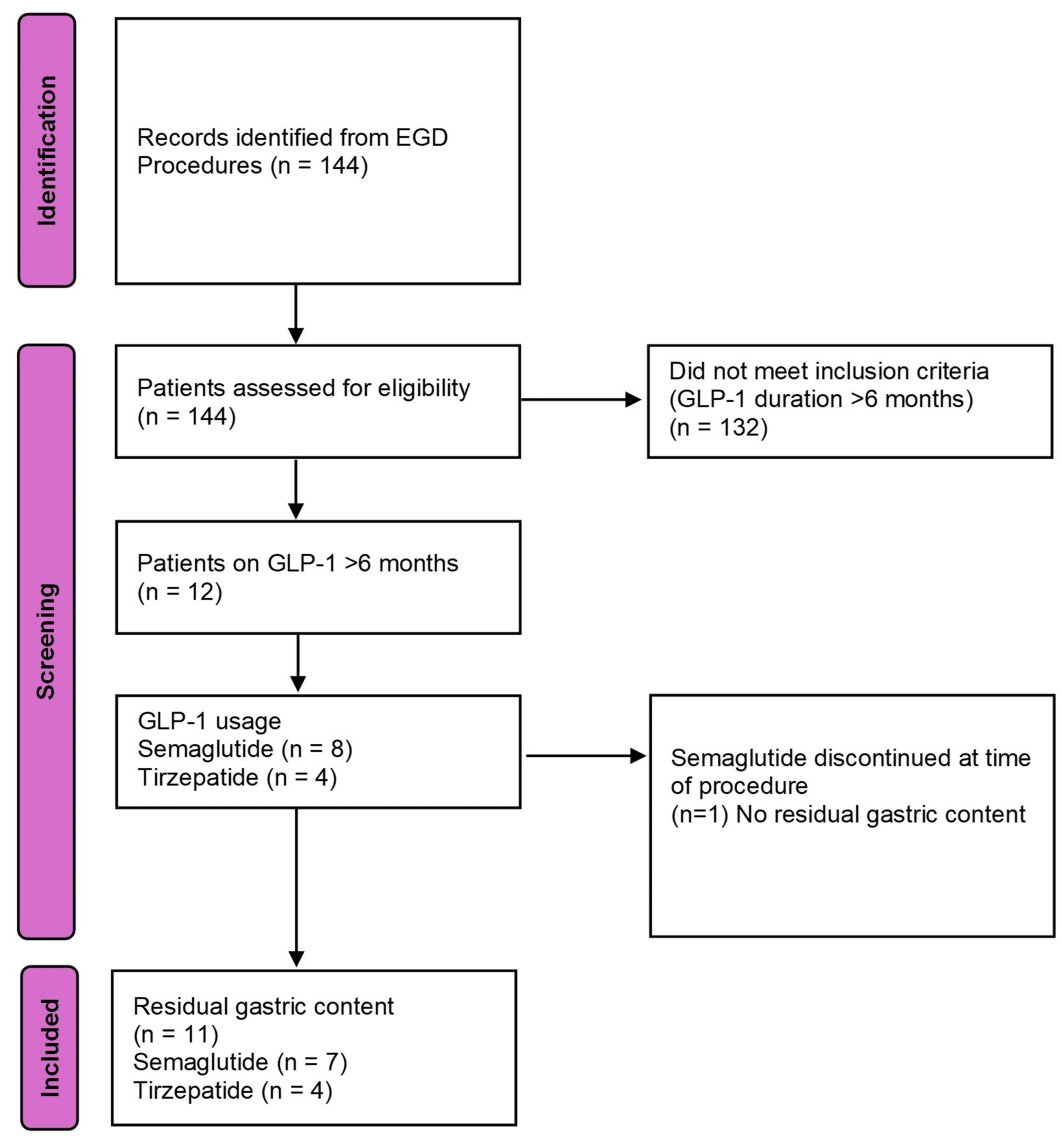
Selection of Patients on Semaglutide or Tirzepatide for Greater Than Six Months Undergoing EGD Evaluated for Residual Gastric Content This flowchart depicts the patient identification, screening, and inclusion for this retrospective cohort study. EGD: esophagogastroduodenoscopy, GLP-1: glucagon-like peptide-1

A total of 144 EGDs performed by a single surgeon (EF) were retrospectively reviewed. Of these, 12 patients were on a GLP-1 agonist for over six months, with the selection process detailed in Figure 1.

Out of the 144 patients in total, the average age was 40±20 years, with 109 female patients and 35 male patients. The average age of the 12 patients was 45±15 years. Gender distribution included 10 female patients and two male patients. The BMI ranged between 42 and 52 kg/m^2^. All patients had gastroesophageal reflux disease and arthritis, and were on continuous positive airway pressure (CPAP) machines for obstructive sleep apnea. Patient demographics are shown in Table 1.

**Table 1:** Included Patients’ Characteristics This table depicts the demographic baseline characteristics of the included patients regarding age, body mass index, presence of gastroesophageal reflux disease, arthritis, and continuous positive airway pressure usage.

Characteristics	Number
Age (years)	45±15
Body mass index (kg/m^2^)	42-52
Gastroesophageal reflux disease	12
Arthritis	12
Continuous positive airway pressure usage	12

Out of the 12 patients, 11 had food particles described in their stomachs during the procedure. In some cases (five patients), the stomach was full of food, making it difficult to visualize the body of the stomach until reaching the antrum. Among the 11 patients with RGC, seven were taking semaglutide (2.4 mg once a day), while the remaining four were taking tirzepatide (2.5 mg). One who had been prescribed semaglutide was not taking it at the time of the procedure, and no food particles were found in their stomach during the EGD. All patients underwent EGDs without complications or aspiration and were discharged on the same day. Upon review of the 132 patient charts that did not fulfill our patient criteria, there were no descriptions of gastroparesis or RGC. Fischer’s exact test was used to determine if there was an association between GLP-1 usage greater than or less than six months. There was a statistically significant association with greater than six months of usage (p<0.001). These findings are found in Table 2.

**Table 2:** Endoscopic Findings of GLP-1 Usage of Less Than and Over Six Months This table stratifies the usage of GLP-1 for greater than and less than six months of therapy with respect to the presence of residual gastric content. GLP-1: glucagon-like peptide-1

Endoscopic findings	GLP-1 usage of <6 months (number)	GLP-1 usage of >6 months (number)	p-value
Normal	132 (100%)	1 (8.3%)	p<0.001
Residual gastric content	0 (0%)	11 (91.7%)	

Effects of age or BMI

Exploring the relationship between RGC with age or BMI, Spearman’s correlation suggested a moderate positive correlation between RGC and age, but a weak negative association with BMI (Table 3).

**Table 3:** Spearman’s Rank Correlation This table presents Spearman’s rank correlation coefficients between residual gastric content and age/body mass index.

Variable	Spearman’s rho	p-value
Age (years)	0.48	0.114
Body mass index (kg/m^2^)	-0.22	0.495

Firth’s logistic regression demonstrated good model fit, with a McFadden’s pseudo-R² of 0.943. However, the individual predictors (GLP-1 type, age, and BMI) were not statistically significant. These findings are found in Table 4.

**Table 4:** Firth’s Logistic Regression This table summarizes the Firth’s logistic regression model that predicts residual gastric content during endoscopy for patients taking a GLP-1. The independent variables assessed were age, sex, and body mass index. This model was chosen to account for the small sample size in this study. GLP-1: glucagon-like peptide-1

Variable	Coefficient	p-value
GLP-1 type (semaglutide/tirzepatide)	-4.622	0.324
Age	1.368	0.390
Body mass index	-1.020	0.412

## Discussion

This retrospective cohort study included 12 patients on a semaglutide or tirzepatide for greater than six months, with 11 having findings of RGC despite standard pre-EGD fasting procedures. None of the 132 patients who were on semaglutide or tirzepatide for less than six months had findings of RGC. This suggests a potential relationship between longer-term GLP-1 usage and RGC, raising important considerations for clinicians when advising patients on pre-procedure evaluation and fasting protocols.

GLP-1 RA usage has increased in popularity over recent years, resulting in more patients presenting for procedures/surgery while taking these medications [7]. GLP-1 RAs exhibit different mechanisms of action depending on whether they are long- or short-acting. Short-acting agents such as exenatide and lixisenatide function primarily by delaying gastric emptying to decrease post-prandial hyperglycemia [6]. Long-acting agents such as semaglutide, liraglutide, dulaglutide, and exenatide (extended-release) increase insulin secretion and suppress glucagon levels to modulate post-prandial hyperglycemia. Short-acting GLP-1 RAs also decrease gastric emptying to a greater degree compared to longer-acting GLP-1 RAs [6]. However, in the case-control study by Kobori et al. of 205 pairs, there was greater RGC in the GLP-1 RA treatment group versus control (0.49% versus 5.4%, p=0.004) [8]. Among the patients taking GLP-1 RAs with RGC, they were prescribed long-acting versions (liraglutide, dulaglutide, and semaglutide) [8]. Hjerpsted et al. noted that there was a notable delay in gastric emptying in the first hour [9]. Thus, the possibility of RGC among long-acting GLP-1 RAs must still be considered. Another consideration for delayed gastric emptying is that patients may have diabetic gastroparesis at baseline, given that semaglutide is traditionally used for type 2 diabetes [8].

Despite strictly adhering to the preoperative fasting guidelines set by the ASA, EGD revealed food residue in the gastric body, leading to the decision to abort the procedure to reduce the risk of intraoperative pulmonary aspiration. This occurrence highlights the absence of preoperative fasting guidelines specific to patients on semaglutide and the delayed gastric emptying known to occur with its use [7]. Anesthesiologists should be aware of alternative methods to ensure an empty stomach in these patients, to mitigate the risk of pulmonary aspiration during general anesthesia [7].

In the study by Dahl et al., 15 participants (mean age: 58.2 years, HbA1c: 6.9%, body weight: 93.9 kg, diabetes duration: 3.1 years, males: 86.7%) were evaluated [10]. Fasting glucose levels were significantly lower, and C-peptide levels were significantly higher with oral semaglutide compared to placebo. Post-prandial glucose (AUC0-5h) was significantly reduced with semaglutide (estimated treatment ratio: 0.71, 95% confidence interval (CI): 0.63-0.81; p<0.0001), as well as glucose incremental AUC (iAUC0-5h/5h) and glucagon AUC0-5h. Similar effects were seen after a fat-rich meal. Additionally, fasting triglycerides, very low-density lipoprotein (VLDL), and apolipoprotein B48 (ApoB48) concentrations were significantly lower with semaglutide versus placebo. In the first post-prandial hour, gastric emptying was delayed by 31% (measured via paracetamol AUC0-1h) in the semaglutide group. One serious adverse event (acute myocardial infarction) occurred during oral semaglutide treatment [10].

Perez-Montes et al. highlighted the rise of obesity as a public health issue and the growing need for effective treatments [11]. While bariatric surgery is the most effective treatment for morbid obesity, less aggressive options are desirable. GLP-1 receptor agonists, such as semaglutide, have emerged as effective treatments for obesity by promoting satiety and delaying gastric emptying. Liraglutide (3 mg) is currently the only FDA-approved GLP-1 RA for obesity, showing weight loss of up to 8.5 kg in short periods. This review underscores the potential of GLP-1 receptor agonists, including oral semaglutide, in the treatment of obesity and diabetes [11].

The study by Jensterle et al. found that semaglutide increased the retention of gastric contents at one, two, three, and four hours after ingestion of a radiolabeled solid meal, with 37% of the meal retained in the stomach at four hours compared to no retention in the placebo group (p=0.002) [12]. The time for half of the radiolabeled meal to empty from the stomach was significantly longer in the semaglutide group (171 minutes) compared to the placebo (118 minutes; p<0.001).

The study by Aldhaleei et al. assessed the GI side effects of GLP-1 receptor agonists in a real-world cohort [13]. Among 10,328 new GLP-1 RA users, the mean age was 61.4±12.6 years (65.7% female patients and 51.3% White). Common GI adverse events included abdominal pain (57.6%), constipation (30.4%), and nausea/vomiting (23.4%) [13]. Semaglutide had lower rates of adverse GI events compared to dulaglutide and liraglutide, but it was still associated with some GI side effects, including gastroparesis (5.1%) [13]. However, gastroparesis with semaglutide was significantly lower compared to dulaglutide, liraglutide, and exenatide [13]. Importantly, GLP-1 RAs, including semaglutide, should be prescribed with caution in patients with preexisting GI issues, and further studies are needed to evaluate the safety of GLP-1 RAs alongside concomitant medications.

The study by Wu et al. reviewed EGDs performed between 2019 and 2023 and compared the incidence of RGC between patients on GLP-1 agonists (90 procedures) and those who began therapy within 1,000 days post-EGD (102 procedures) [14]. The GLP-1 cohort had a higher incidence of RGC (19%) compared to the control group (5%), with a confounder-adjusted odds ratio of 5.8 (95% CI: 1.7-19.3; p=0.004). There were also more instances of emergent intubation (five versus one) and pulmonary aspiration (one versus none) in the GLP group. This suggests that GLP-1 therapy increases the risk of RGC in fasting patients, potentially leading to periprocedural pulmonary aspiration [14].

In the case report by Chaudhry et al., a patient developed gastroparesis after starting semaglutide [1]. This highlights the potential for semaglutide to exacerbate GI symptoms, such as bloating, nausea, and abdominal discomfort, and underlines the importance of monitoring for GI side effects in patients. Similarly, the case report by Klein et al., where a patient started weekly semaglutide injections two months earlier, found substantial gastric content during EGD despite having fasted for 18 hours [15].

The study by Silveira et al. similarly supported delayed gastric emptying and increased RGC among patients who received semaglutide within 30 days of their EGD [16]. However, 404 EGDs were included in their analysis, with 33 taking semaglutide and 371 non-semaglutide. Of these, eight (24.2%) patients taking semaglutide and 19 (5.1%) patients taking non-semaglutide had increased RGC [16].

This study provides additional evidence of the need for greater precaution for patients taking semaglutide and other GLP-1 RAs. Suggestions such as placing patients on a liquid diet 36 hours before an EGD, adding prokinetic drugs such as metoclopramide or erythromycin, more conservative fasting recommendations, and adding preoperative ultrasound evaluation of gastric contents have been raised [7]. RGC is one of the biggest predisposing factors for pulmonary aspiration. Although a rare event, pulmonary aspiration can lead to significant morbidity and mortality [7]. Furthermore, patients undergoing procedures/surgery under general anesthesia commonly use anesthetic agents such as propofol or opioids, which carry an increased risk of aspiration from depression of the lower esophageal sphincter, causing loss of the protective airway reflex [7]. Additionally, the use of CPAP, which is common among patients with obesity or obstructive sleep apnea, can lead to transient gastric insufflation secondary to continuous airflow into the upper airway [17]. Although this insufflation is temporary until CPAP discontinuation, these patients share overlapping risk factors for aspiration and regurgitation during anesthesia.

However, several limitations must be addressed. First, the limited sample size for inclusion decreases the generalizability of these findings and prohibits a determination of causal effect despite the statistical significance. Studies with a greater sample size are required to support these findings. Second, retrospective studies introduce selection bias as these patients are undergoing endoscopies for specific indications, which may not represent the broader cohort of GLP-1 RA users. Third, there was no control group to determine if the RGC observed was due to GLP-1 RA usage.

## Conclusions

Patients who have been on GLP-1 RA for six months or longer show a correlation with increased RGC in 91.7% of our cases, albeit with a small sample size. This represents a significant concern for anesthesia and surgical teams, as these patients are at higher risk for aspiration. Their therapeutic effects are, at least in part, mediated by their influence on gastric emptying. However, despite being NPO for 12 hours or more, all participants had food in their stomachs. This study highlights the attention to detail needed toward RGC among patients taking semaglutide and tirzepatide undergoing an EGD. As a result, we have revised our practice to recommend stopping GLP-1 RAs one week before EGDs.
